# Natural Killer Cells and Current Applications of Chimeric Antigen Receptor-Modified NK-92 Cells in Tumor Immunotherapy

**DOI:** 10.3390/ijms20020317

**Published:** 2019-01-14

**Authors:** Jianguang Zhang, Huifang Zheng, Yong Diao

**Affiliations:** School of Medicine, Huaqiao University, Quanzhou 362021, Fujian, China; 1601116007@hqu.edu.cn (J.Z.); 15136733530@163.com (H.Z.)

**Keywords:** natural killer cell line, NK-92, chimeric antigen receptor, immunotherapy, tumor

## Abstract

Natural killer (NK) cells are innate immune cells that can be activated rapidly to target abnormal and virus-infected cells without prior sensitization. With significant advancements in cell biology technologies, many NK cell lines have been established. Among these cell lines, NK-92 cells are not only the most widely used but have also been approved for clinical applications. Additionally, chimeric antigen receptor-modified NK-92 cells (CAR-NK-92 cells) have shown strong antitumor effects. In this review, we summarize established human NK cell lines and their biological characteristics, and highlight the applications of NK-92 cells and CAR-NK-92 cells in tumor immunotherapy.

## 1. Introduction

Natural killer (NK) cells are innate immune cells that were first discovered in mice in 1975 [1]. NK cells account for approximately 10% of lymphocytes in human peripheral blood (PB). Owing to the distinct chemokine receptors expressed in NK cells, the distributions of NK cells differ among healthy tissues. Most NK cells are found in the PB, liver, spleen, and bone marrow, and a small portion are also present in the lymph nodes [2,3,4,5]. NK cells are part of the first line of defense that protects the body from pathogen invasion and malignant transformation. When normal cells are infected by viruses, NK cells are rapidly activated to protect against abnormal and virus-infected cells, without prior sensitization [5,6,7].

In recent studies, as our understanding of tumor immunology has deepened, the basic research and clinical applications of NK cells has become an interesting topic. Some studies have shown that allogeneic NK cells have stronger tumor killing ability than autologous NK cells [8]. Moreover, allogeneic NK cells can be obtained from many sources, such as bone marrow, human embryonic stem cells, induced pluripotent stem cells, PB, and umbilical cord blood. However, these NK cells are difficult to purify and expand in vitro. With the gradual advancement of cell cloning technology, many NK cell lines have been established, including KHYG-1, NK-92, NKL, NKG, and YT cells. All of these cell lines are characterized by a uniform phenotype, high purity, and function, consistent with the general characteristics of NK cells. These cells can also be cultured at a large scale in vitro, thus providing sufficient cells for research and clinical applications. Among these cell lines, NK-92 cells are the most widely used line that have been approved for clinical use. Additionally, chimeric antigen receptor (CAR)-modified NK-92 (CAR-NK-92) cells have shown strong antitumor effects.

In this review, we summarize the established human NK cell lines and their biological characteristics and highlight the applications of NK-92 cells and CAR-NK-92 cells in tumor immunotherapy.

## 2. Receptor Distribution and Killing Mechanism of NK Cells

**Figure 1:**
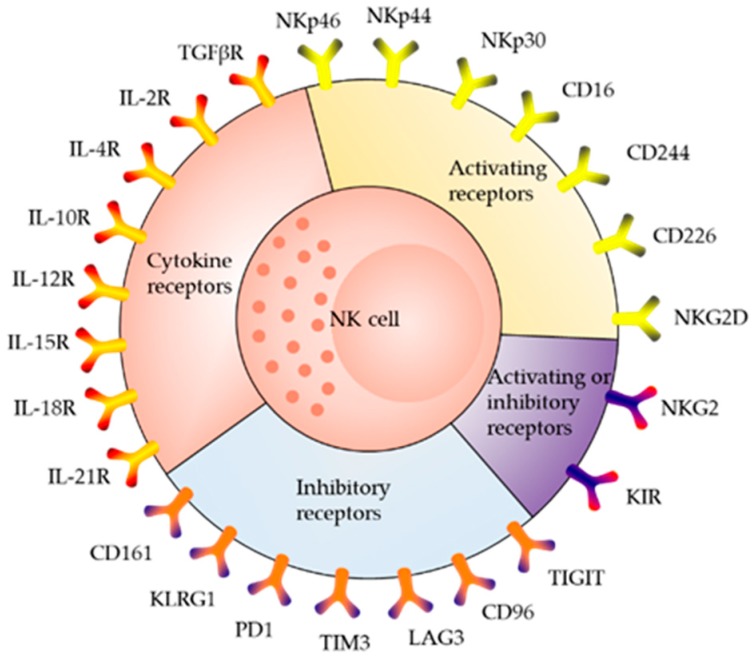
Major receptors expressed on the surface of natural killer (NK) cells. NKp46, natural killer cell p46-related protein; NKp44, natural killer cell p44-related protein; NKp30, natural killer cell p30-related protein; CD, Cluster of differentiation; NKG2, also known as CD159; KIR, killer-cell immunoglobulin-like receptor; TIGIT, T cell immunoreceptor with Ig and ITIM domains; LAG3, lymphocyte activation gene 3 protein; TIM3, T cell immunoglobulin mucin receptor 3; PD1, programmed cell death protein 1; KLRG1, killer cell lectin-like receptor subfamily G member 1; IL-2R, interleukin-2 receptor; TGFβR, transforming growth factor beta receptors.

As the body’s first line of defense, many surface molecules (Figure 1) are expressed on NK cells [9,10], which are characterized by the expression of CD56 and CD16 cell surface markers, and absence of T-cell receptor (TCR) and B-cell receptor. According to differences in CD56 and CD16 expression density, two major subsets of NK cells can be distinguished, i.e., CD56bright and CD56dim [3,5,11,12]. CD56dim NK cells are fully mature, account for approximately 90% of the NK cells in the PB, and predominantly mediate cytotoxicity responses [5]. CD56dim cells can play roles in antibody-dependent cell-mediated cytotoxicity (ADCC) through the surface expression of CD16 (FcγRIII) [13,14,15]. CD56bright cells are immature, account for approximately 5–15% of the NK cells in the PB, and are predominant in tissues and secondary lymphoid organs [16]. CD56bright cells express high levels of CD56, CD94/NKG2A, l-selectin, and a high-affinity interleukin (IL)-2 receptor, but little to no CD16 and killer-cell immunoglobulin-like receptor. Therefore, CD56bright cells function primarily to secrete cytokines, such as interferon-γ, tumor necrosis factor (TNF)-α, and granulocyte macrophage-colony-stimulating factor [11,17].

NK cells do not express antigen-specific recognition receptors. There are two receptors with opposite functions on their surface. The first type of receptor can bind to its corresponding ligand on the surface of the target cell, activating the killing effects of NK cells. This receptor is called the activating NK cell receptor [18]. The other type of receptor, called the inhibitory NK cell receptor, inhibits the killing effect of NK cells [18]. 

Both the activating receptor and inhibitory receptor can recognize classical or nonclassical major histocompatibility complex (MHC) class I molecules expressed on the surface of normal cells. Inhibitory receptors play a primary role in preventing NK cells from killing normal cells [19,20]. In virus-infected cells and tumor cells, MHC class I molecules on the cell surface are lost or downregulated; activating NK cell receptors play a leading role in this process. 

**Figure 2:**
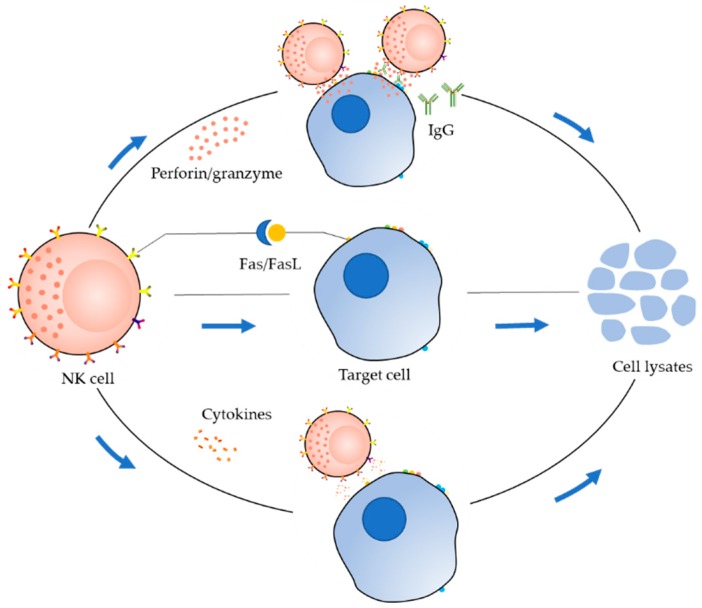
Mechanisms of cytotoxicity by natural killer (NK) cells.

Activated NK cells exert cytotoxic effects mainly through three pathways (Figure 2). The first pathway is the perforin/granzyme pathway [9]. In this pathway, granzymes and perforin stored in the cytoplasmic granules of NK cells are released by activated NK cells together into the intercellular space. The structure of perforin is similar to that of complement, which can form a transmembrane channel directly on the target cell membrane, resulting in increased permeability of the cell membrane and ultimately causing osmotic lysis of the target cell. The perforation channel formed by perforin facilitates the entry of granzyme into the target cell. Additionally, perforin causes redistribution of granzymes in the cytoplasm and nucleus of target cells, allowing granzymes to accumulate at the cleavage site; this promotes the lysis of target cells and ultimately apoptosis. The second pathway is the Fas/FasL pathway [21,22]. The Fas molecule, also known as Apo-1 or CD95, is a type-Ⅰ transmembrane protein, and Fas ligand (FasL or CD95L) is a type-Ⅱ transmembrane protein that belongs to the TNF family. When FasL binds to Fas, Fas can deliver a “death signal” to the cell, and the target cell then undergoes apoptosis within a few hours. In the third pathway, the cytokine pathway, NK cells secrete cytokines, e.g., TNF-α [9], which can alter the stability of lysosomes in target cells, leading to leakage of various hydrolases, affecting cell membrane phospholipid metabolism, and activating target cell endonuclease to degrade genomic DNA.

## 3. Currently Known NK Cell Lines

NK cell lines are monoclonal cells that can survive permanently in vitro. Here, we summarize currently established primary NK cell lines (Table 1).

### 3.1. NK3.3 Cells

NK3.3 cells are a normal NK-derived cell line obtained by Kornbluth and co-workers in 1982 [23]. These cells are dependent on Interleukin-2 (IL-2) and are positive for CD2, CD11a, CD38, CD45, CD16, and CD56 [24]. The morphology, immunohistochemistry, and phenotype of NK3.3 cells are similar to those of large granular lymphocytes (LGLs). NK3.3 cells have very strong cytotoxicity, which can kill NK-sensitive target cells, such as K562 and MOLT-4 cells [23,25,26].

### 3.2. YT Cells

The YT cell line was established in 1983 by Yodoi and co-workers, derived from a 15-year-old pericardial effusion of a Japanese man suffering from acute lymphoma and thymoma [27]. YT cells show varying sizes, irregular nuclei, and abundant vacuoles, and azurophilic particles in the cytoplasm. Additionally, YT cells are positive for CD56 and negative for CD2, CD3, and CD16. Importantly, YT cells can undergo long-term in vitro expansion without the need for conditioned medium or IL-2. Moreover, YT cells have toxic effects on cancer cells, such as K562, MOLT-4, HPB-ALL, and HSB-2 cells. 

YT2C2 and YTC3 cells are two subclones of YT cells [28]. These two cell subclones do not mediate ADCC. However, these cell lines can kill 721.221 cells with high expression of B7.1 [29]. Cytotoxicity analysis showed that YT2C2 and YTC3 cells primarily rely on cell surface receptor-mediated cytotoxicity.

### 3.3. NKL Cells

The NKL cell line was established in 1996 by Robertson and co-workers and was derived from the PB of a patient with LGL leukemia [30]. NKL cells require IL-2 for sustained growth and die if deprived of IL-2 for more than 7 days. The morphology of NKL cells is similar to that of activated NK cells, which have natural killing activity and can mediate ADCC. According to phenotypic analysis, NKL cells overexpress CD2, CD6, CD11a, CD27, CD29, CD38, CD43, CD58, CD94, and CD95, but are negative for CD3, CD4, CD5, CD8, CD14, CD19, CD20, and CD28. As the culture time in vitro increased, the cell surface expression of CD16, CD56, and CD57 and the antigen density were significantly reduced.

### 3.4. HANK1 Cells

The HANK1 cell line was established in 1998 by Kagami and co-workers [31]. This cell line was derived from a 46-year-old woman with CD56^+^ NK/T-cell lymphoma. HANK1 cells are large polymorphic cells with irregular nuclei and azurophilic granules in the cytoplasm. Immunophenotypic analysis showed that HANK1 cells were positive for CD2, CD3ε, CD56, and CD25. Moreover, HANK1 cells exhibit IL-2 dependence during in vitro culture and retain the biological characteristics of the original tumor cells, allowing them to be used as a model of abnormal nasal-type NK/T-cell lymphoma [31].

### 3.5. NK-YS Cells

The NK-YS cell line was established by Tsuchiyama and co-workers in 1996 [32]. This cell line was derived from a 19-year-old woman with leukemic-state nasal angiocentric NK cell lymphoma with systemic skin infiltration. Immunophenotypic analysis showed that NK-YS cells retain the characteristics of prototypic NK lymphoma cells and positively express CD2, CD5, CD7, and CD56, but are negative for CD3, CD16, and CD57. 

### 3.6. KHYG-1 Cells

The KHYG-1 cell line was established by Yagita and co-workers in 1997 and was derived from a 45-year-old woman with aggressive NK cell leukemia [33]. KHYG-1 cells have the morphological characteristics of LGLs, i.e., large nuclei, rough chromosomes, obvious nucleoli, abundant cytoplasm, basophilic features, and azurophilic granules. Additionally, these cells rely on IL-2 during in vitro culture. The immunophenotype of KHYG-1 cells is as follows: positive for CD2, CD3ε, CD7, CD8αα, CD33, CD56, CD122, and CD132, and negative for CD1, CD3, CD16, CD25, CD34, and CD57. Suck and co-workers [34] found that the KHYG-1 cell line is more cytotoxic to K562 cells and leukemia cells lines, such as EM2, EM3, and HL60. Additionally, KHYG-1 cells are most likely to regulate cell lysis with perforin by granzyme M (instead of granzymes A and B). Researchers have predicted that KHYG-1 may induce apoptosis in tumor cells through a novel granzyme/perforin pathway. 

### 3.7. SNK-6 and SNT-8 Cells

SNK-6 and SNT-8 cells are nasal NK/T-cell lymphoma cells established in 2001 by Nagata and co-workers in Japan [35]. SNK-6 cells were derived from a 62-year-old Japanese man, and SNT-8 cells were derived from a 48-year-old Japanese woman. These two cell lines were established using a single-cell suspension culture method. Tumor cells from male patients were cultured for 20 months and named SNK-6, and tumor cells from female patients were cultured for 17 months and named SNT-8. Both cell lines rely on IL-2 for growth. Immunophenotypic analysis showed that SNK-6 cells are positive for CD56 and negative for CD3, CD4, CD8, CD19, and TCR (TCRα/β and TCR γ/δ), whereas SNT-8 cells are positive for CD3, CD56, and TCRγ/δ, and negative for CD4, CD8, CD19, and TCRα/β. These two cell lines have been shown to have important applications in studies of cell necrosis caused by nasal T/NK cell lymphoma.

### 3.8. IMC-1 Cells

The IMC-1 cell line was established in 2004 by Chen and colleagues [36]. These cells are an IL-2-dependent leukemia cell line obtained from an adult patient with invasive NK cell leukemia. IMC-1 cells express CD56, CD2, CD11a, CD38, and HLA-DR cell surface antigens and lyse target cells in a non-MHC-restricted and antibody-independent manner.

### 3.9. NK-92 Cells

The NK-92 cell line was isolated and successfully established by Klingemann’s group in 1992 from a 50-year-old man with malignant non-Hodgkin’s lymphoma [37]. NK-92 cells express CD2 and CD56 and are negative for CD3, CD4, CD8, and CD16. The growth of NK-92 cells is dependent on the presence of recombinant IL-2; once IL-2 is withdrawn, the cells will die within 72 h. NK-92 cells tend to grow in an aggregated pattern and easily die when dispersed [24,37]. Similar to NK cells, NK-92 cells can kill tumor cells without prior sensitization. Additionally, NK-92 cells lack the CD16 receptor and therefore cannot mediate ADCC. However, these cells still exhibit a high degree of cytotoxic activity toward a variety of cancers cells [38,39,40]. NK-92MI and NK-92CI cells are derived from NK-92 cells and have biological characteristics similar to those of NK-92 cells, but do not rely on IL-2 [38].

## 4. Progress in the Application of NK-92 Cells

Among the several NK cell lines currently established, NK-92 cells lack almost all inhibitory killing receptors and express a series of activating receptors [42]. Additionally, NK-92 cells are abundant in perforin and granzyme, suggesting a broad spectrum of cytotoxic effects. Accordingly, these cells have become a critical NK cell line for preclinical research and are the only NK cell line approved by the US Food and Drug Administration (FDA) for phase I and II clinical studies [43,44,45,46,47].

After the establishment of NK-92 cells, many studies were carried out to demonstrate the potential applications of NK-92 cells in the treatment of tumors. Yan and co-workers [43,44,45,46,47,48] studied the cell-killing effects of NK-92 cells on primary tumor cells derived from various leukemia patients using chromium 51 release tests. The results showed that NK-92 cells were cytotoxic to leukemia cells both in vitro and in vivo. Swift and co-workers studied the cytotoxicity of NK-92 cells to bulk and clonogenic multiple myeloma and evaluated the effects of NK-92 cell treatment in a xenograft mouse model. The results demonstrated that NK-92 cells could effectively kill clonogenic and bulk multiple myeloma cells and could significantly reduce tumor burden in vivo [49]. 

The growth of NK-92 cells is dependent on the presence of IL-2; however, systemic administration of IL-2 can result in unexpected side effects, such as high toxicity and nonlocalized administration [50]. To make NK-92 cells more suitable for clinical applications, Konstantinidis and co-workers used gene editing to express IL-2 in the endoplasmic reticulum of NK-92 cells; the engineered NK-92 cells could grow normally independent of IL-2 and exhibited the same function and cytotoxicity as NK-92 cells [50]. 

In combination with current good manufacturing practice (cGMP)-compliant expansion methodologies [51], NK-92 cells are approved for analysis in clinical trials to determine their utility in the treatment of some types of malignant tumors [47,52]. For example, Tam and co-workers [51] demonstrated that when NK-92 cells were irradiated with 500 cGy gamma rays, the proliferation of NK-92 cells was prevented, and their high killing activity was maintained. Moreover, even when the irradiation dose was increased to 1000 cGy, the cytotoxic effects of NK-92 cells were maintained within 48 h. Additionally, Tonn and co-workers [47] conducted a comprehensive study of the possible limitations of NK-92 cells in clinical applications and determined the optimal culture conditions and operating procedures for large-scale expansion under GMP conditions. Subsequently, a phase Ⅰ trial was performed in seven patients with advanced cancer; the NK-92 cells were infused twice per patient with an interval of 48 h, and individual patients developed low-heat, back pain, and other uncomfortable reactions after treatment. Generally, it was found to be safe to infuse 5 × 10^9^/m^2^ of NK-92 cells per patient. Further experiments are needed to determine the efficacy of NK-92 cells in advanced cancer [47]. 

## 5. Structure of CARs and Their Applications in NK-92 Cells

In recent studies, major breakthroughs in the use of CAR-T cells for relapsed and refractory B cell malignancies targeting CD19 have been made [53,54,55,56,57,58,59,60,61,62], and two CD19 CAR-T cell products have been approved by the US FDA [63,64].

However, there are many limitations to the use of CAR-T cells, including off-target effects and cytokine storms. Therapy with CAR-T cells has not yet been successful in patients with solid tumors, and the production of autologous cells also has many limitations in the clinical setting [65]. The production of CAR-T cells requires a certain time period, which makes it challenging to prepare a sufficient number of CAR-T cells within a short time for patients whose disease progresses faster. It is also difficult to collect a sufficient number of healthy T cells from the patient. “Off-the-shelf” allogeneic T cells can overcome these difficulties, but may cause severe graft-versus-host disease (GVHD) [66]. 

CAR-NK-92 cells are highly cytotoxic and can be harvested as phenotypically homogeneous cells, with production of large numbers of cells within a short period. Additionally, compared with CAR-T cells, CAR-NK-92 cells cannot proliferate after irradiation; thus, the survival time in vivo is shorter, avoiding some off-target effects [67]. Even if the targeted antigen on the tumor is rapidly lost, the CAR-NK-92 cells can still be activated by their activating receptors, conferring CAR-NK-92 cells with significant advantages.

### 5.1. Structure of CARs

**Figure 3:**
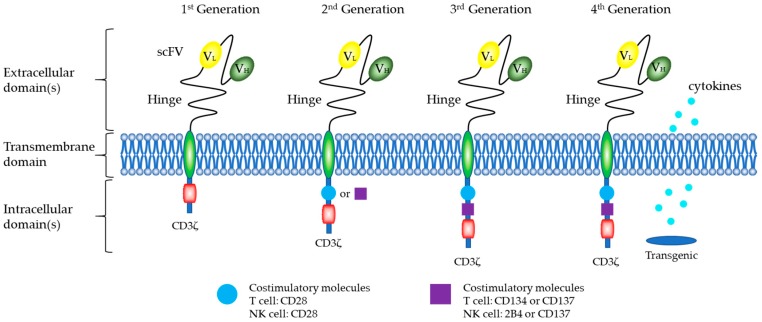
Generations of CAR design. The traditional CAR vector structure consists of three parts: an extracellular antigen recognition region, a transmembrane region, and an intracellular signal domain. The extracellular domain of CAR includes an scFv region (H [heavy] and L [light] chain) that is spliced by a linker. A hinge ensures flexibility and connects to the transmembrane domain. The intracellular domain includes a CD3ζ signaling domain and costimulatory domains, such as CD28, CD134, CD137, and 2B4.

The traditional CAR vector structure consists of three parts (Figure 3), including an extracellular antigen recognition region, a transmembrane region, and intracellular signal domain, which determine the specificity and functionality of the CAR modification. Current CAR technology is in the fourth generation of development [68]. The first generation of CARs consisted of a single-chain variable fragment (scFv) that recognizes tumor surface antigens, and an immunoreceptor tyrosine-based activation motif (ITAM, usually CD3ζ) [69]. However, the first generation of CARs only caused T cells to proliferate for a short period and did not provide long-term T-cell expansion signals to maintain antitumor effects.

According to the dual-signal model of T-cell activation, the second and third generations of CARs introduced CD28, CD134 (OX40), CD137 (4-1BB) [70], and other costimulatory molecules. The aim was to enhance the antitumor cytotoxicity and proliferative capacity of T cells in vivo.

To more completely remove tumor cells, Chmielewski and co-workers [71,72] designed the “fourth generation” CAR structure. The fourth generation of CARs contains two costimulatory molecules (CD28, CD134, or CD137) and induces the secretion of IL-12 from the cell. In the treatment of solid cancer, fourth-generation CAR-T cells release IL-2 and activate innate immune cells to eliminate antigen-negative cancer cells, thereby increasing the antitumor effect of these cells in vivo by several fold.

#### CARs of NK Cells

Similar to CAR-T cells, the goal of CAR-NK cells is to establish a new activation pathway to enhance the antitumor effects of the cells and to improve tumor cell targeting. CAR-NK have the basic framework of CAR-T (Figure 3), including an extracellular antigen recognition region, a transmembrane region, and an intracellular signal domain.

CD3ζ is a classical intracellular signal segment of the CAR structure [73] and plays an important role in NK cells [74]. CAR-NK generally uses CD3ζ as the first signal motif (first-generation CAR) and then a costimulatory molecular motif (second-generation CAR), such as CD28 or CD137 (4-1BB) [75], to form an intracellular signal region.

NKG2D is a crucial activating receptor expressed on most CD8^+^ T cells and NK cells and is a relatively unique activating receptor in NK cells. The NKG2D receptor binds to DAP10 or DAP12 transfer proteins to provide different activation signals [76]. Both signals can activate the cytotoxicity of NK cells, but only the activation signal transmitted by DAP12 can promote the production of cytokines by NK cells [77]. In one study [78], researchers linked DAP10 and CD3ζ to the NK cell activation receptor NKG2D. In an osteosarcoma mouse model, the cytotoxic potential of NK cells against a wide spectrum of tumor subtypes could be markedly enhanced by expression of CAR-NKG2D-DAP10-CD3ζ receptor. 

CD244, also known as the NK cell receptor 2B4, is a signal transduction lymphocyte-activating molecule-related receptor expressed in all NK cells [79]. This protein is an important regulator of NK cell activation and was shown to have robust costimulatory roles in a study in which NK cells were used as effector cells to target CD19 or GD2 [80].

### 5.2. Preclinical Studies of CAR-NK-92 Cells

NK-92 cells are an ideal CAR carrier with natural antitumor properties and are easy to cultivate and modify in vitro. The first generation of CAR has been widely applied in CAR-NK-92 cells (Table 2) [16]. 

Uherek and co-workers [81] reported the expression of an antigen recognition receptor on the surface of NK-92 cells by gene editing. This receptor recognizes the tumor-associated antigen ErbB2, which is overexpressed in various epithelial tumors. Moreover, they used a first-generation technique similar to CAR-T cells. The entire CAR structure included the extracellular region ErbB2-specific scFv (FRP5) antibody fragment, a CD8 hinge region, a transmembrane region, and an intracellular CD3ζ chain. In vitro experiments indicated that compared with the parental cells, the genetically modified NK-92-scFv (FRP5)-CD3ζ cells were able to specifically recognize and effectively kill ErbB2-expressing tumor cells from different sources. Subsequently, CAR-NK-92, the first-generation CAR structure of different targets, showed good effects against hematomas and solid tumors. This structure also included CD19 and CD20 for B cell leukemia and lymphoma [82,83,84,85], CD138 for myeloma [86], human epidermal growth factor receptor 2 (HER2) and epidermal growth factor receptor for brain metastasis [87,88,89], HER2 and EGFR for breast cancer [81,90,91,92,93,94], and GD2 for neuroblastoma [95,96]. Additionally, NK-92 cells do not express CD16 and therefore do not mediate the effects of ADCC. In recent years, methods for expressing CD16 have been established, and expression of CD16 in NK-92 cells can effectively enhance the antitumor effects of the cells [97]. Boissel and co-workers demonstrated that CAR-NK-92 cells have the ability to clear chronic lymphocytic leukemia cells to a greater extent than NK-92 cells expressing CD16 [85].

The efficacy and safety of CAR-NK-92 cells based on second- and third-generation CARs (Table 3) were also confirmed in preclinical trials. 

In one study, Oelsner and co-workers [83] compared the cytotoxic effects of CAR-CD19-CD3ζ-NK-92, CAR-CD19-CD28-CD3ζ-NK-92, and CAR-CD19-CD137-CD3ζ-NK-92 on established B-cell leukemia and lymphoma cells. The results showed that all three CD19-specific CAR-NK-92 cell lines were effective at killing B cell malignancies. However, CAR-CD19-CD137-CD3ζ-NK-92 cells were less effective than CAR-CD19-CD3ζ-NK-92 and CAR-CD19-CD28-CD3ζ-NK-92 cells at cell killing and cytokine production, indicating the differential effects of the costimulatory CD28 and CD137 domains. In a recent study [98], researchers at the University of California, San Diego evaluated the effects of different CAR constructs on NK cell-mediated killing. They designed nine CAR constructs that target mesothelin. The killing experiments in vitro revealed that a CAR containing the transmembrane domain of NKG2D, the 2B4 costimulatory domain, and the CD3ζ signaling domain of CAR could mediate strong antigen-specific NK cell signaling. Subsequently, the effects of CAR-NK and CAR-T cells were compared in a mouse model. The results showed that CAR-NK cells had in vivo activities similar to those of as CAR-T cells, but with less toxicity. This research indicated that CAR-NK cell therapy may be safer than CAR-T cell therapy.

### 5.3. Ongoing Clinical Trials

Clinical trials examining CAR-NK-92 cells for the treatment of tumors are being carried out (Table 4). However, compared with CAR-T cells that have been applied in clinical studies, fewer clinical studies have been performed for CAR-NK-92 cells [8,99], and little clinical data have been published. 

In a recent clinical trial (NCT02944162), Tang and co-workers [100] revealed the efficacy and safety of CAR-NK-92 cells in the treatment of relapsed and refractory acute myeloid leukemia. In this study, a total of three patients received therapy with CD33-CAR NK-92 cells. The first patient was a 14-year-old girl and the second patient was a 24-year-old male, who both received doses of 3 × 10^8^, 6 × 10^8^, and 1 × 10^9^ cells on days 1, 3, and 5, respectively. Another patient was a 49-year-old woman who received doses of 1 × 10^9^, 3 × 10^9^, and 5 × 10^9^ cells on days 1, 4 and 7, respectively. All patients had mild symptoms after treatment, including fever and cytokine release syndrome, but returned to normal the next day. This is the first phase Ⅰ clinical trial of CD33-CAR NK-92 cells in patients with relapsed and refractory acute myeloid leukemia. Although the treatment effect was not significant, the findings of this clinical study showed that CAR-NK-92 cells can be safely used.

### 5.4. Advantages of CAR-NK-92 Cells

CAR-NK cells are potential competitors for CAR-T cells. CAR-NK-92 cells do not cause GVHD [111] and have greater cytotoxicity than ADCC [85]. Indeed, CAR-NK-92 cells have many advantages, as follows: (1) CAR-NK-92 cells can target tumor cells and directly activate NK-92 cells to kill target cells; (2) even if the targeted antigen on the tumor is rapidly lost, the CAR-NK-92 cells can still be activated by their activating receptors [8]; (3) the inhibitory receptors are expressed at low levels on the surface [37] and deletion of inhibitory receptors makes NK-92 cells more resistant to solid tumors than other immune cells; and (4) NK-92 cells are immortalized cell lines with a uniform phenotype, allowing them to be cultured in vitro for a long time to proliferate. CAR-NK-92 cells also have this advantage, which can compensate for the decline in immune cell viability in patients with advanced cancer. Depending on the type of tumor, corresponding CAR-NK-92 cells can be directly expanded for treatment, thereby shortening the treatment cycle and reducing the cost of treatment [112].

### 5.5. Challenges and Coping Strategies

Although CAR-NK-92 cells have good antitumor effects, there are still some problems that need to be solved to make them more suitable for clinical treatment.

#### 5.5.1. Tumor-Producing and Potential Epstein-Barr (EB) Virus Susceptibility

NK-92 cells are derived from patients with malignant non-Hodgkin’s lymphoma; they may cause secondary tumorigenesis and potential EB virus susceptibility after injection. Therefore, for safety reasons, NK-92 cells need to be lethally irradiated before clinical application [16].

#### 5.5.2. NK-92 Cells Have A Short Life Cycle after Irradiation

After irradiation, CAR-NK-92 cells survive in vivo for a short period. This method reduces the side effects of CAR-NK-92 cells on the body, while also reducing the antitumor effects; thus, therapy may require multiple injections.

#### 5.5.3. Defects in the Transfected Vector

Genetic modification of cells is a critical step to realizing CAR expression. Virus transduction is the most common method used for genetic modification. These viral vectors include retroviral vectors, lentiviral vectors, and adenoviral vectors, among which retroviral vectors and lentiviral vectors are most widely used. Viral vectors are capable of ensuring stable expression of the transgene. However, the viral vector itself may be related to high production costs, a long reproduction cycle, and complexity. Additionally, viral vectors alone cannot accurately insert genes into the desired location, posing potential risks; for example, they may affect the expression of normal genes or cause the inserted genes to be abnormally regulated. Accordingly, it is essential to develop non-viral vectors. The CRISPR-Cas9 system can be combined with electroporation techniques to precisely insert genes into the genome and achieve stable expression. Roth and co-workers [113] successfully demonstrated that the system can successfully edit the genome at low cost and complete T-cell modification in just a few weeks.

#### 5.5.4. Off-Target Effects

CAR-NK-92 cells are target dependent and mainly kill cells with high expression of specific antigens. However, once the antigens are also expressed in normal tissues and cells, off-target toxicity can occur. Currently, there are no specific clinical data to assess the severity of the off-target toxicity of CAR-NK-92 cells. Accordingly, it is necessary to consider dose control and other factors (e.g., introducing suicide genes) in order to reduce the risk of toxicity [114].

## 6. Conclusions and Perspectives

The antitumor ability of NK cells confers them with broad potential applications in cell therapy. NK cells modified by CAR have also been shown to be promising as effector cells. However, the exploration of CAR-NK cells is still at a preliminary stage. It is necessary to examine which types of CAR structures are most effective for activating NK cells and which intracellular signal domains can maintain the killing ability of NK cells for a long time. Although currently used viral vectors can be stably integrated into the genome, there is a risk of random insertion. Thus, development of new non-viral transfection methods is necessary. NK-92 cells, an immortalized cell line, can be quickly and easily obtained in the clinical setting. Additionally, the cells must be subjected to lethal radiation before use in order to avoid the risk of secondary tumorigenicity. Controllable NK-92 cells, such as those expressing a combination of suicide genes, should be further explored. With advancements in CAR-NK technology and the accumulation of clinical experience, combination of CAR-NK cells with other anticancer therapies may also show efficacy. Although CAR-NK cells induce a low incidence of cytokine storms and induce few side effects compared with CAR-T cells, further studies are needed to fully assess the safety of these cells. As preclinical trials and clinical studies proceed, NK cells are expected to play important roles in the treatment of cancer.

## Figures and Tables

**Table 1:** Currently known NK cell lines.

Cell Line	Year	Disease Diagnosis	Patient	Doubling Time	Viral Status	Cytokine	Primary Reference
NK3.3	1982	NR	NR	NR	EBV^−^	IL-2-dependent	[23]
YT	1983	Acute lymphoblastic lymphoma (with thymoma)	15-year-old male	40–50 h	EBV^+^	Independent of IL-2	[27]
NKL	1996	NK-LGLL	63-year-old male	24–48 h	NR	IL-2-dependent	[30,41]
HANK1	1998	Nasal-like NK/T-cell lymphoma	46-year-old female	3 day	EBV^+^	IL-2-dependent	[31]
NK-YS	1996	NK cell lymphoma, Nasal angiocentric, Leukemic state with systemic skin infiltration	19-year-old female	48 h	EBV^+^	IL-2-dependent	[32]
KHYG-1	1997	Aggressive NK leukemia	45-year-old female	24–48 h	EBV^−^	IL-2-dependent	[33]
SNK-6	1998	Nasal NK/T-cell lymphoma	62-year-old male	NR	EBV^+^	IL-2-dependent	[35]
SNT-8	1998	Nasal NK/T-cell lymphoma	48-year-old female	NR	EBV^+^	IL-2-dependent	[35]
IMC-1	2004	Aggressive NK cell leukemia	42-year-old male	24–36 h	EBV^−^	IL-2-dependent	[36]
NK-92	1992	LGL-NHL	50-year-old male	24 h	EBV^−^	IL-2-dependent; Growth stimulation:IL-7	[37]

NR, not reported; EBV, Epstein-Barr virus; NK, natural killer cell; LGL: large granular lymphocyte; LGLL: large granular lymphocyte leukemia; NHL: non-Hodgkin’s lymphoma.

**Table 2:** Preclinical studies with the first generation of CAR-NK-92 cells.

Cancer Type	Antigen Targeted	Hinge	TM	Intracellular Signal Domain	Genetic Modification Method	Effector Cell	Year	References
Multiple myeloma	CD138	CD8	CD3ζ	CD3ζ	lentiviral vector	NK-92MI	2014	[86]
B-cell malignancies	CD19	CD8	NR	CD3ζ	Retrovirus	NK-92	2016	[82]
B-cell malignancies	CD19	CD8	CD28	CD3ζ	Lentiviral	NK-92	2017	[83]
CLL	CD19	CD8	CD3ζ	CD3ζ	Electroporation	NK-92	2009	[84]
ALL CLL	CD19 CD20	NR	NR	CD3ζ	Lentivirus	NK-92	2014	[85]
B-cell malignancies	CD20	CD8	CD3ζ	CD3ζ	Retroviral	NK-92	2008	[101]
Prostate cancer	EpCAM	CD8	CD3ζ	CD3ζ	Retrovirus	NK-92	2009	[102]
Prostate cancer	EpCAM	CD8	CD3ζ	CD3ζ	Retrovirus	NK-92	2011	[103]
Neuroblastoma	GD2	CD8	CD3ζ	CD3ζ	Retrovirus	NK-92	2012	[96]
Neuroblastoma	GD2	CD8	CD3ζ	CD3ζ	Retrovirus	NK-92	2015	[95]
Melanoma	GPA7	NR	HLA-A2	CD3ζ	Electroporation	NK-92MI	2013	[104]
Brain metastasis	HER2	CD8α	CD3ζ	CD3ζ	Retrovirus	NK-92	2016	[88]
Brain metastasis	HER2	CD8	CD3ζ	CD3ζ	Retrovirus	NK-92	2013	[89]
Breast cancer	HER2	CD8	CD3ζ	CD3ζ	Retrovirus	NK-92	2005	[91]
Breast cancer	HER2	CD8	CD3ζ	CD3ζ	Retrovirus	NK-92	2008	[92]
Breast/ovarian cancer	HER2	CD8	CD3ζ	CD3ζ	Retrovirus	NK-92	2002	[81]
Breast cancer, Ovarian cancer, Melanoma Renal cell carcinoma	HER2	CD8	CD3ζ	CD3ζ	Lentiviral	NK-92	2015	[93]
Ovarian cancer Mesothelin-expressing tumors	Mesothelin	CD8	NKG2D	CD3ζ	Transposon plasmids	NK-92	2018	[98]

**Table 3:** Preclinical studies with the second and third generations of CAR-NK-92 cells.

Cancer Type	Antigen Targeted	Hinge	TM	Intracellular Signal Domain	Genetic Modification Method	Effector Cell	Year	References
B-cell malignancies	CD19	CD8	CD28	CD28-CD3ζ CD137-CD3ζ	Lentiviral	NK-92	2017	[83]
Multiple myeloma	CS1	NR	NR	CD28-CD3ζ	Lentivirus	NK-92	2014	[105]
EBV^+^ cells	EBNA3C	NR	NR	CD137-CD3ζ	Retrovirus	NK-92MI	2012	[75]
Glioblastoma	EGFR EGFRvIII	NR	CD28	CD28-CD3ζ	Lentivirus	NK-92 and NKL	2015	[106]
Brain metastasis	EGFR	NR	NR	CD28-CD3ζ	Lentivirus	NK-92	2016	[87]
Glioblastoma	EGFR EGFRvIII	CD8	CD28	CD28-CD3ζ	Lentivirus	NK-92	2015	[107]
Breast cancer	EpCAM	CD8	CD28	CD28-CD3ζ	Lentivirus	NK-92	2012	[90]
Breast cancer Renal cell carcinoma Ovarian carcinoma Melanoma	HER2	CD8	CD28 CD137	CD28-CD3ζ CD137-CD3ζ	Lentiviral	NK-92	2015	[93]
Glioblastoma	HER2	CD8	CD28	CD28-CD3ζ	Lentiviral	NK-92	2016	[108]
Breast cancer	HER2	CD8	CD28	CD28-CD3ζ	Electroporation	NK-92	2015	[94]
Ovarian cancer Mesothelin-expressing tumors	Mesothelin	CD8	CD16	2B4-CD3ζ	Transposon plasmids	NK-92	2018	[98]
Ovarian cancer mesothelin-expressing tumors	Mesothelin	CD8	NKp44	DAP10-CD3ζ 2B4-CD3ζ	Transposon plasmids	NK-92	2018	[98]
Ovarian cancer Mesothelin-expressing tumors	Mesothelin	CD8	NKG2D	2B4-CD3ζ CD137-CD3ζ	Transposon plasmids	NK-92	2018	[98]
Ovarian cancer Mesothelin-expressing tumors	Mesothelin	CD8	CD28	CD28-CD137-CD3ζ	Transposon plasmids	NK-92	2018	[98]
Ovarian cancer Mesothelin-expressing tumors	Mesothelin	CD8	NKG2D	2B4-DAP12-CD3ζ 2B4-DAP10-CD3ζ CD137-2B4-CD3ζ	Transposon plasmids	NK-92	2018	[98]
Aggressive T cell malignancies	CD3	CD8	CD8	CD28-CD137-CD3ζ	Lentivirus	NK-92	2016	[109]
Aggressive T-cell malignancies	CD5	CD8	CD8	CD28-CD137-CD3ζ	Lentivirus	NK-92	2017	[110]

NR, not reported; ALL, acute lymphoblastic leukemia; B-ALL, B-cell acute lymphoblastic leukemia; CLL, chronic lymphocytic leukemia; EGFR, epidermal growth factor receptor; HER2, human epidermal growth factor receptor 2; TM, transmembrane domain; TAMs, tumor-associated macrophages.

**Table 4:** Clinical trials with CAR-NK-92 cells.

NCT Number	NK Cell Source	Target Antigen	Disease	Phase	Estimated Enrollment	Age	Location	References
NCT02742727	NK-92	CD7	Acute Myeloid Leukemia;Precursor T-Cell Lymphoblastic Leukemia-Lymphoma; T-cell Prolymphocytic Leukemia; T-cell Large Granular Lymphocytic Leukemia; Peripheral T-cell Lymphoma, NOS; Angioimmunoblastic T-cell Lymphoma Extranodal NK/T-cell Lymphoma, Nasal Type; Enteropathy-type Intestinal T-cell Lymphoma; Hepatosplenic T-cell Lymphoma	Phase 1 Phase 2	10 participants	18 Years and older (Adult, Older Adult)	China	NR
NCT02892695	NK-92	CD19	Acute Lymphocytic Leukemia; Chronic Lymphocytic Leukemia; Follicular Lymphoma; Mantle Cell Lymphoma; B-cell Prolymphocytic Leukemia; Diffuse Large Cell Lymphoma;	Phase 1 Phase 2	10 participants	3 Years to 80 Years (Child, Adult, Older Adult)	China	NR
NCT02944162	NK-92	CD33	Acute Myelogenous Leukemia; Acute Myeloid Leukemia; Acute Myeloid Leukemia with Maturation; Acute Myeloid Leukemia Without Maturation; ANLL	Phase 1 Phase 2	10 participants	3 Years to 80 Years (Child, Adult, Older Adult)	China	[100]
NCT03383978	NK-92	HER2	Glioblastoma	Phase 1	30 participants	18 Years and older (Adult, Older Adult)	Germany	NR
NCT03656705	NK-92	NR	Non-small Cell Lung Cancer	Phase 1	5 participants	18 Years to 75 Years (Adult, Older Adult)	China	NR

NR, not reported; Data are from http://www.clinicaltrials.gov.

## Abbreviations

ADCC	Antibody dependent cell mediated cytotoxicity
CAR	Chimeric antigen receptor
cGMP	Current good manufacturing practice
EB	Epstein-Barr
FDA	Food and Drug Administration
GVHD	Graft-versus-host disease
HER2	Human epidermal growth factor receptor 2
IL-2	Interleukin-2
ITAMs	Immunoreceptor tyrosine-based activation motif
KIRs	Killer immunoglobulin-like receptor
LGLs	Large granular lymphocytes
MHC	Major histocompatibility complex
NK	Natural killer
PB	Peripheral blood
scFv	Single-chain variable fragment
TCR	T-cell receptor
TNF	Tumor necrosis factor

## References

[1] Fang F., Xiao W., Tian Z. (2017). NK cell-based immunotherapy for cancer. Semin. Immunol..

[2] Hazenberg M.D., Spits H. (2014). Human innate lymphoid cells. Blood.

[3] Caligiuri M.A. (2008). Human natural killer cells. Blood.

[4] Rezvani K., Rouce R., Liu E., Shpall E. (2017). Engineering Natural Killer Cells for Cancer Immunotherapy. Mol. Ther..

[5] Campbell K.S., Hasegawa J. (2013). Natural killer cell biology: An update and future directions. J. Allergy Clin. Immun..

[6] Cerwenka A., Lanier L.L. (2016). Natural killer cell memory in infection, inflammation and cancer. Nat. Rev. Immunol..

[7] Hammer Q., Ruckert T., Romagnani C. (2018). Natural killer cell specificity for viral infections. Nat. Immunol..

[8] Mehta R.S., Rezvani K. (2018). Chimeric Antigen Receptor Expressing Natural Killer Cells for the Immunotherapy of Cancer. Front. Immunol..

[9] Chiossone L., Dumas P.Y., Vienne M., Vivier E. (2018). Natural killer cells and other innate lymphoid cells in cancer. Nat. Rev. Immunol..

[10] Handgretinger R., Lang P., André M.C. (2016). Exploitation of natural killer cells for the treatment of acute leukemia. Blood.

[11] Romagnani C., Juelke K., Falco M., Morandi B., D’Agostino A., Costa R., Ratto G., Forte G., Carrega P., Lui G. (2007). CD56brightCD16- Killer Ig-Like Receptor- NK Cells Display Longer Telomeres and Acquire Features of CD56dim NK Cells upon Activation. J. Immunol..

[12] Fehniger T.A., Cooper M.A., Nuovo G.J., Cella M., Facchetti F., Colonna M., Caligiuri M.A. (2003). CD56bright natural killer cells are present in human lymph nodes and are activated by T cell-derived IL-2: A potential new link between adaptive and innate immunity. Blood.

[13] Clynes R.A., Towers T.L., Presta L.G., Ravetch J.V. (2000). Inhibitory Fc receptors modulate in vivo cytotoxicity against tumor targets. Nat. Med..

[14] Cooper M.A., Fehniger T.A., Caligiuri M.A. (2001). The biology of human natural killer-cell subsets. Trends Immunol..

[15] Romain G., Senyukov V., Rey-Villamizar N., Merouane A., Kelton W., Liadi I., Mahendra A., Charab W., Georgiou G., Roysam B. (2014). Antibody Fc engineering improves frequency and promotes kinetic boosting of serial killing mediated by NK cells. Blood.

[16] Lin C., Zhang J. (2018). Reformation in chimeric antigen receptor based cancer immunotherapy: Redirecting natural killer cell. Biochim. Biophys. Acta Rev. Cancer.

[17] De Maria A., Bozzano F., Cantoni C., Moretta L. (2011). Revisiting human natural killer cell subset function revealed cytolytic CD56(dim)CD16+ NK cells as rapid producers of abundant IFN-gamma on activation. Proc. Natl. Acad. Sci. USA.

[18] Lanier L.L. (2008). Up on the tightrope: Natural killer cell activation and inhibition. Nat. Immunol..

[19] Sivakumar P.V., Gunturi A., Salcedo M., Schatzle J.D., Lai W.C., Kurepa Z., Pitcher L., Seaman M.S., Lemonnier F.A., Bennett M. (1999). Cutting edge: Expression of functional CD94/NKG2A inhibitory receptors on fetal NK1.1+Ly-49- cells: A possible mechanism of tolerance during NK cell development. J. Immunol..

[20] Kumar S. (2018). Natural killer cell cytotoxicity and its regulation by inhibitory receptors. Immunology.

[21] Wajant H. (2002). The Fas Signaling Pathway: More Than a Paradigm. Science.

[22] Waring P., Mullbacher A. (1999). Cell death induced by the Fas/Fas ligand pathway and its role in pathology. Immunol. Cell Biol..

[23] Kornbluth J., Flomenberg N., Dupont B. (1982). Cell surface phenotype of a cloned line of human natural killer cells. J. Immunol..

[24] Le Bouteiller P., Barakonyi A., Giustiniani J., Lenfant F., Marie-Cardine A., Aguerre-Girr M., Rabot M., Hilgert I., Mami-Chouaib F., Tabiasco J. (2002). Engagement of CD160 receptor by HLA-C is a triggering mechanism used by circulating natural killer (NK) cells to mediate cytotoxicity. Proc. Natl. Acad. Sci. USA.

[25] Umehara H., Huang J.Y., Kono T., Tabassam F.H., Okazaki T., Bloom E.T., Domae N. (1997). Involvement of protein tyrosine kinase p72syk and phosphatidylinositol 3-kinase in CD2-mediated granular exocytosis in the natural killer cell line, NK3.3. J. Immunol..

[26] Mahle N.H., Radcliff G., Sevilla C.L., Kornbluth J., Callewaert D.M. (1989). Kinetics of cellular cytotoxicity mediated by a cloned human natural killer cell line. Immunobiology.

[27] Yodoi J., Teshigawara K., Nikaido T., Fukui K., Noma T., Honjo T., Takigawa M., Sasaki M., Minato N., Tsudo M. (1985). TCGF (IL 2)-receptor inducing factor(s). I. Regulation of IL 2 receptor on a natural killer-like cell line (YT cells). J. Immunol..

[28] Yoneda N., Tatsumi E., Kawano S., Teshigawara K., Oka T., Fukuda M., Yamaguchi N. (1992). Detection of Epstein-Barr virus genome in natural-killer-like cell line, YT. Leukemia.

[29] Chen X., Allan D., Krzewski K., Ge B., Kopcow H., Strominger J.L. (2006). CD28-stimulated ERK2 phosphorylation is required for polarization of the microtubule organizing center and granules in YTS NK cells. Proc. Natl. Acad. Sci. USA.

[30] Robertson M.J., Cochran K.J., Cameron C., Le J.M., Tantravahi R., Ritz J. (1996). Characterization of a cell line, NKL, derived from an aggressive human natural killer cell leukemia. Exp. Hematol..

[31] Kagami Y., Nakamura S., Suzuki R., Iida S., Yatabe Y., Okada Y., Kobayashi T., Tsurumi T., Seto M., Ogura M. (1998). Establishment of an IL-2-dependent cell line derived from ‘nasal-type’ NK/T-cell lymphoma of CD2+, sCD3-, CD3epsilon+, CD56+ phenotype and associated with the Epstein-Barr virus. Br. J. Haematol..

[32] Tsuchiyama J., Yoshino T., Mori M., Kondoh E., Oka T., Akagi T., Hiraki A., Nakayama H., Shibuya A., Ma Y. (1998). Characterization of a novel human natural killer-cell line (NK-YS) established from natural killer cell lymphoma/leukemia associated with Epstein-Barr virus infection. Blood.

[33] Yagita M., Huang C.L., Umehara H., Matsuo Y., Tabata R., Miyake M., Konaka Y., Takatsuki K. (2000). A novel natural killer cell line (KHYG-1) from a patient with aggressive natural killer cell leukemia carrying a p53 point mutation. Leukemia.

[34] Suck G., Branch D.R., Smyth M.J., Miller R.G., Vergidis J., Fahim S., Keating A. (2005). KHYG-1, a model for the study of enhanced natural killer cell cytotoxicity. Exp. Hematol..

[35] Nagata H., Konno A., Kimura N., Zhang Y., Kimura M., Demachi A., Sekine T., Yamamoto K., Shimizu N. (2001). Characterization of novel natural killer (NK)-cell and gammadelta T-cell lines established from primary lesions of nasal T/NK-cell lymphomas associated with the Epstein-Barr virus. Blood.

[36] Chen I., Whalen M., Bankhurst A., Sever C.E., Doshi R., Hardekopf D., Montgomery K., Willman C.L. (2004). A new human natural killer leukemia cell line, IMC-1. A complex chromosomal rearrangement defined by spectral karyotyping: Functional and cytogenetic characterization. Leukemia Res..

[37] Gong J.H., Maki G., Klingemann H.G. (1994). Characterization of a human cell line (NK-92) with phenotypical and functional characteristics of activated natural killer cells. Leukemia.

[38] Tam Y.K., Maki G., Miyagawa B., Hennemann B., Tonn T., Klingemann H.G. (1999). Characterization of genetically altered, interleukin 2-independent natural killer cell lines suitable for adoptive cellular immunotherapy. Hum. Gene Ther..

[39] Tam Y.K., Miyagawa B., Ho V.C., Klingemann H.G. (1999). Immunotherapy of malignant melanoma in a SCID mouse model using the highly cytotoxic natural killer cell line NK-92. J. Hematother..

[40] Klingemann H.G., Miyagawa B. (1996). Purging of malignant cells from blood after short ex vivo incubation with NK-92 cells. Blood.

[41] Isobe Y., Sugimoto K., Yang L., Tamayose K., Egashira M., Kaneko T., Takada K., Oshimi K. (2004). Epstein-Barr virus infection of human natural killer cell lines and peripheral blood natural killer cells. Cancer Res..

[42] Maki G., Klingemann H.G., Martinson J.A., Tam Y.K. (2001). Factors regulating the cytotoxic activity of the human natural killer cell line, NK-92. J. Hematother. Stem Cell. Res..

[43] Boyiadzis M., Agha M., Redner R.L., Sehgal A., Im A., Hou J., Farah R., Dorritie K.A., Raptis A., Lim S.H. (2017). Phase 1 clinical trial of adoptive immunotherapy using “off-the-shelf” activated natural killer cells in patients with refractory and relapsed acute myeloid leukemia. Cytotherapy.

[44] Tonn T., Schwabe D., Klingemann H.G., Becker S., Esser R., Koehl U., Suttorp M., Seifried E., Ottmann O.G., Bug G. (2013). Treatment of patients with advanced cancer with the natural killer cell line NK-92. Cytotherapy.

[45] Arai S., Meagher R., Swearingen M., Myint H., Rich E., Martinson J., Klingemann H. (2008). Infusion of the allogeneic cell line NK-92 in patients with advanced renal cell cancer or melanoma: A phase I trial. Cytotherapy.

[46] Williams B.A., Law A.D., Routy B., DenHollander N., Gupta V., Wang X.H., Chaboureau A., Viswanathan S., Keating A. (2017). A phase I trial of NK-92 cells for refractory hematological malignancies relapsing after autologous hematopoietic cell transplantation shows safety and evidence of efficacy. Oncotarget.

[47] Tonn T., Becker S., Esser R., Schwabe D., Seifried E. (2001). Cellular immunotherapy of malignancies using the clonal natural killer cell line NK-92. J. Hematother. Stem Cell. Res..

[48] Yan Y., Steinherz P., Klingemann H.G., Dennig D., Childs B.H., McGuirk J., O’Reilly R.J. (1998). Antileukemia activity of a natural killer cell line against human leukemias. Clin. Cancer Res..

[49] Swift B.E., Williams B.A., Kosaka Y., Wang X.H., Medin J.A., Viswanathan S., Martinez-Lopez J., Keating A. (2012). Natural killer cell lines preferentially kill clonogenic multiple myeloma cells and decrease myeloma engraftment in a bioluminescent xenograft mouse model. Haematologica.

[50] Konstantinidis K.V., Alici E., Aints A., Christensson B., Ljunggren H.G., Dilber M.S. (2005). Targeting IL-2 to the endoplasmic reticulum confines autocrine growth stimulation to NK-92 cells. Exp. Hematol..

[51] Tam Y.K., Martinson J.A., Doligosa K., Klingemann H.G. (2003). Ex vivo expansion of the highly cytotoxic human natural killer-92 cell-line under current good manufacturing practice conditions for clinical adoptive cellular immunotherapy. Cytotherapy.

[52] Klingemann H., Boissel L., Toneguzzo F. (2016). Natural Killer Cells for Immunotherapy—Advantages of the NK-92 Cell Line over Blood NK Cells. Front. Immunol..

[53] Pegram H.J., Smith E.L., Rafiq S., Brentjens R.J. (2015). CAR therapy for hematological cancers: Can success seen in the treatment of B-cell acute lymphoblastic leukemia be applied to other hematological malignancies?. Immunotherapy.

[54] Porter D.L., Hwang W.T., Frey N.V., Lacey S.F., Shaw P.A., Loren A.W., Bagg A., Marcucci K.T., Shen A., Gonzalez V. (2015). Chimeric antigen receptor T cells persist and induce sustained remissions in relapsed refractory chronic lymphocytic leukemia. Sci. Transl. Med..

[55] Lee D.W., Kochenderfer J.N., Stetler-Stevenson M., Cui Y.K., Delbrook C., Feldman S.A., Fry T.J., Orentas R., Sabatino M., Shah N.N. (2015). T cells expressing CD19 chimeric antigen receptors for acute lymphoblastic leukaemia in children and young adults: A phase 1 dose-escalation trial. Lancet.

[56] Davila M.L., Riviere I., Wang X., Bartido S., Park J., Curran K., Chung S.S., Stefanski J., Borquez-Ojeda O., Olszewska M. (2014). Efficacy and toxicity management of 19-28z CAR T cell therapy in B cell acute lymphoblastic leukemia. Sci. Transl. Med..

[57] Maude S.L., Frey N., Shaw P.A., Aplenc R., Barrett D.M., Bunin N.J., Chew A., Gonzalez V.E., Zheng Z., Lacey S.F. (2014). Chimeric antigen receptor T cells for sustained remissions in leukemia. N. Engl. J. Med..

[58] Sredni B., Longo D.L. (2012). Cancer immunotherapy: Are we there yet?. Semin. Cancer Biol..

[59] Porter D.L., Levine B.L., Kalos M., Bagg A., June C.H. (2011). Chimeric antigen receptor-modified T cells in chronic lymphoid leukemia. N. Engl. J. Med..

[60] Kochenderfer J.N., Wilson W.H. (2010). Eradication of B-lineage cells and regression of lymphoma in a patient treated with autologous T cells genetically engineered to recognize CD19. Blood.

[61] Brentjens R.J., Davila M.L., Riviere I., Park J., Wang X., Cowell L.G., Bartido S., Stefanski J., Taylor C., Olszewska M. (2013). CD19-targeted T cells rapidly induce molecular remissions in adults with chemotherapy-refractory acute lymphoblastic leukemia. Sci. Transl. Med..

[62] Grupp S.A., Kalos M., Barrett D., Aplenc R., Porter D.L., Rheingold S.R., Teachey D.T., Chew A., Hauck B., Wright J.F. (2013). Chimeric antigen receptor-modified T cells for acute lymphoid leukemia. N. Engl. J. Med..

[63] (2018). FDA Approves Second CAR T-cell Therapy. Cancer Discov..

[64] (2017). First-ever CAR T-cell therapy approved in U.S. Cancer Discov..

[65] Wang Z., Wu Z., Liu Y., Han W. (2017). New development in CAR-T cell therapy. J. Hematol. Oncol..

[66] Jacoby E., Yang Y., Qin H., Chien C.D., Kochenderfer J.N., Fry T.J. (2016). Murine allogeneic CD19 CAR T cells harbor potent antileukemic activity but have the potential to mediate lethal GVHD. Blood.

[67] Zhang C., Oberoi P., Oelsner S., Waldmann A., Lindner A., Tonn T., Wels W.S. (2017). Chimeric Antigen Receptor-Engineered NK-92 Cells: An Off-the-Shelf Cellular Therapeutic for Targeted Elimination of Cancer Cells and Induction of Protective Antitumor Immunity. Front. Immunol..

[68] Fan M., Li M., Gao L., Geng S., Wang J., Wang Y., Yan Z., Yu L. (2017). Chimeric antigen receptors for adoptive T cell therapy in acute myeloid leukemia. J. Hematol. Oncol..

[69] Jensen M.C., Riddell S.R. (2014). Design and implementation of adoptive therapy with chimeric antigen receptor-modified T cells. Immunol. Rev..

[70] Wang J., Jensen M., Lin Y., Sui X., Chen E., Lindgren C.G., Till B., Raubitschek A., Forman S.J., Qian X. (2007). Optimizing adoptive polyclonal T cell immunotherapy of lymphomas, using a chimeric T cell receptor possessing CD28 and CD137 costimulatory domains. Hum. Gene Ther..

[71] Chmielewski M., Abken H. (2015). TRUCKs: The fourth generation of CARs. Expert Opin. Biol. Ther..

[72] Chmielewski M., Kopecky C., Hombach A.A., Abken H. (2011). IL-12 release by engineered T cells expressing chimeric antigen receptors can effectively Muster an antigen-independent macrophage response on tumor cells that have shut down tumor antigen expression. Cancer Res..

[73] Love P.E., Hayes S.M. (2010). ITAM-mediated signaling by the T-cell antigen receptor. Cold Spring Harb. Perspect. Biol..

[74] Arnon T.I., Markel G., Mandelboim O. (2006). Tumor and viral recognition by natural killer cells receptors. Semin. Cancer Biol..

[75] Tassev D.V., Cheng M., Cheung N.K. (2012). Retargeting NK92 cells using an HLA-A2-restricted, EBNA3C-specific chimeric antigen receptor. Cancer Gene Ther..

[76] Gilfillan S., Ho E.L., Cella M., Yokoyama W.M., Colonna M. (2002). NKG2D recruits two distinct adapters to trigger NK cell activation and costimulation. Nat. Immunol..

[77] Trinchieri G. (2003). The choices of a natural killer. Nat. Immunol..

[78] Chang Y.H., Connolly J., Shimasaki N., Mimura K., Kono K., Campana D. (2013). A chimeric receptor with NKG2D specificity enhances natural killer cell activation and killing of tumor cells. Cancer Res..

[79] McNerney M.E., Lee K., Kumar V. (2005). 2B4 (CD244) is a non-MHC binding receptor with multiple functions on natural killer cells and CD8+ T cells. Mol. Immunol..

[80] Altvater B., Landmeier S., Pscherer S., Temme J., Schweer K., Kailayangiri S., Campana D., Juergens H., Pule M., Rossig C. (2009). 2B4 (CD244) Signaling by Recombinant Antigen-specific Chimeric Receptors Costimulates Natural Killer Cell Activation to Leukemia and Neuroblastoma Cells. Clin. Cancer Res..

[81] Uherek C., Tonn T., Uherek B., Becker S., Schnierle B., Klingemann H.G., Wels W. (2002). Retargeting of natural killer-cell cytolytic activity to ErbB2-expressing cancer cells results in efficient and selective tumor cell destruction. Blood.

[82] Romanski A., Uherek C., Bug G., Seifried E., Klingemann H., Wels W.S., Ottmann O.G., Tonn T. (2016). CD19-CAR engineered NK-92 cells are sufficient to overcome NK cell resistance in B-cell malignancies. J. Cell. Mol. Med..

[83] Oelsner S., Friede M.E., Zhang C., Wagner J., Badura S., Bader P., Ullrich E., Ottmann O.G., Klingemann H., Tonn T. (2017). Continuously expanding CAR NK-92 cells display selective cytotoxicity against B-cell leukemia and lymphoma. Cytotherapy.

[84] Boissel L., Betancur M., Wels W.S., Tuncer H., Klingemann H. (2009). Transfection with mRNA for CD19 specific chimeric antigen receptor restores NK cell mediated killing of CLL cells. Leukemia Res..

[85] Boissel L., Betancur M., Lu W., Krause D., Van Etten R., Wels W., Klingemann H. (2014). Retargeting NK-92 cells by means of CD19- and CD20-specific chimeric antigen receptors compares favorably with antibody-dependent cellular cytotoxicity. Oncoimmunology.

[86] Jiang H., Zhang W., Shang P., Zhang H., Fu W., Ye F., Zeng T., Huang H., Zhang X., Sun W. (2014). Transfection of chimeric anti-CD138 gene enhances natural killer cell activation and killing of multiple myeloma cells. Mol. Oncol..

[87] Chen X., Han J., Chu J., Zhang L., Zhang J., Chen C., Chen L., Wang Y., Wang H., Yi L. (2016). A combinational therapy of EGFR-CAR NK cells and oncolytic herpes simplex virus 1 for breast cancer brain metastases. Oncotarget.

[88] Alkins R., Burgess A., Kerbel R., Wels W.S., Hynynen K. (2016). Early treatment of HER2-amplified brain tumors with targeted NK-92 cells and focused ultrasound improves survival. Neuro-Oncology.

[89] Alkins R., Burgess A., Ganguly M., Francia G., Kerbel R., Wels W.S., Hynynen K. (2013). Focused Ultrasound Delivers Targeted Immune Cells to Metastatic Brain Tumors. Cancer Res..

[90] Sahm C., Schonfeld K., Wels W.S. (2012). Expression of IL-15 in NK cells results in rapid enrichment and selective cytotoxicity of gene-modified effectors that carry a tumor-specific antigen receptor. Cancer Immunol. Immunother..

[91] Daldrup-Link H.E., Meier R., Rudelius M., Piontek G., Piert M., Metz S., Settles M., Uherek C., Wels W., Schlegel J.R. (2005). In vivo tracking of genetically engineered, anti-HER2/neu directed natural killer cells to HER2/neu positive mammary tumors with magnetic resonance imaging. Eur. Radiol..

[92] Meier R., Piert M., Piontek G., Rudelius M., Oostendorp R.A., Senekowitsch-Schmidtke R., Henning T.D., Wels W.S., Uherek C., Rummeny E.J. (2008). Tracking of [18F]FDG-labeled natural killer cells to HER2/neu-positive tumors. Nucl. Med. Biol..

[93] Schonfeld K., Sahm C., Zhang C., Naundorf S., Brendel C., Odendahl M., Nowakowska P., Bonig H., Kohl U., Kloess S. (2015). Selective inhibition of tumor growth by clonal NK cells expressing an ErbB2/HER2-specific chimeric antigen receptor. Mol. Ther..

[94] Liu H., Yang B., Sun T., Lin L., Hu Y., Deng M., Yang J., Liu T., Li J., Sun S. (2015). Specific growth inhibition of ErbB2expressing human breast cancer cells by genetically modified NK92 cells. Oncol. Rep..

[95] Seidel D., Shibina A., Siebert N., Wels W.S., Reynolds C.P., Huebener N., Lode H.N. (2015). Disialoganglioside-specific human natural killer cells are effective against drug-resistant neuroblastoma. Cancer Immunol. Immunother..

[96] Esser R., Müller T., Stefes D., Kloess S., Seidel D., Gillies S.D., Aperlo-Iffland C., Huston J.S., Uherek C., Schönfeld K. (2012). NK cells engineered to express a GD2-specific antigen receptor display built-in ADCC-like activity against tumour cells of neuroectodermal origin. J. Cell. Mol. Med..

[97] Binyamin L., Alpaugh R.K., Hughes T.L., Lutz C.T., Campbell K.S., Weiner L.M. (2008). Blocking NK cell inhibitory self-recognition promotes antibody-dependent cellular cytotoxicity in a model of anti-lymphoma therapy. J. Immunol..

[98] Li Y., Hermanson D.L., Moriarity B.S., Kaufman D.S. (2018). Human iPSC-Derived Natural Killer Cells Engineered with Chimeric Antigen Receptors Enhance Anti-tumor Activity. Cell Stem Cell.

[99] Martín-Antonio B., Suñe G., Perez-Amill L., Castella M., Urbano-Ispizua A. (2017). Natural Killer Cells: Angels and Devils for Immunotherapy. Int J. Mol. Sci..

[100] Tang X., Yang L., Li Z., Nalin A.P., Dai H., Xu T., Yin J., You F., Zhu M., Shen W. (2018). First-in-man clinical trial of CAR NK-92 cells: Safety test of CD33-CAR NK-92 cells in patients with relapsed and refractory acute myeloid leukemia. Am. J. Cancer Res..

[101] Müller T., Uherek C., Maki G., Chow K.U., Schimpf A., Klingemann H., Tonn T., Wels W.S. (2008). Expression of a CD20-specific chimeric antigen receptor enhances cytotoxic activity of NK cells and overcomes NK-resistance of lymphoma and leukemia cells. Cancer Immunol. Immunother..

[102] Tavri S., Jha P., Meier R., Henning T.D., Muller T., Hostetter D., Knopp C., Johansson M., Reinhart V., Boddington S. (2009). Optical imaging of cellular immunotherapy against prostate cancer. Mol. Imaging.

[103] Meier R., Golovko D., Tavri S., Henning T.D., Knopp C., Piontek G., Rudelius M., Heinrich P., Wels W.S., Daldrup-Link H. (2011). Depicting adoptive immunotherapy for prostate cancer in an animal model with magnetic resonance imaging. Magn. Reson. Med..

[104] Zhang G., Liu R., Zhu X., Wang L., Ma J., Han H., Wang X., Zhang G., He W., Wang W. (2013). Retargeting NK-92 for anti-melanoma activity by a TCR-like single-domain antibody. Immunol. Cell Biol..

[105] Chu J., Deng Y., Benson D.M., He S., Hughes T., Zhang J., Peng Y., Mao H., Yi L., Ghoshal K. (2014). CS1-specific chimeric antigen receptor (CAR)-engineered natural killer cells enhance in vitro and in vivo antitumor activity against human multiple myeloma. Leukemia.

[106] Han J., Chu J., Keung C.W., Zhang J., Wang Y., Cohen J.B., Victor A., Meisen W.H., Kim S.H., Grandi P. (2015). CAR-Engineered NK Cells Targeting Wild-Type EGFR and EGFRvIII Enhance Killing of Glioblastoma and Patient-Derived Glioblastoma Stem Cells. Sci. Rep..

[107] Genssler S., Burger M.C., Zhang C., Oelsner S., Mildenberger I., Wagner M., Steinbach J.P., Wels W.S. (2016). Dual targeting of glioblastoma with chimeric antigen receptor-engineered natural killer cells overcomes heterogeneity of target antigen expression and enhances antitumor activity and survival. Oncoimmunology.

[108] Zhang C., Burger M.C., Jennewein L., Genßler S., Schönfeld K., Zeiner P., Hattingen E., Harter P.N., Mittelbronn M., Tonn T. (2016). ErbB2/HER2-Specific NK Cells for Targeted Therapy of Glioblastoma. J. Natl. Cancer Inst..

[109] Chen K.H., Wada M., Firor A.E., Pinz K.G., Jares A., Liu H., Salman H., Golightly M., Lan F., Jiang X. (2016). Novel anti-CD3 chimeric antigen receptor targeting of aggressive T cell malignancies. Oncotarget.

[110] Chen K.H., Wada M., Pinz K.G., Liu H., Lin K.W., Jares A., Firor A.E., Shuai X., Salman H., Golightly M. (2017). Preclinical targeting of aggressive T-cell malignancies using anti-CD5 chimeric antigen receptor. Leukemia.

[111] Domogala A., Madrigal J.A., Saudemont A. (2015). Natural Killer Cell Immunotherapy: From Bench to Bedside. Front. Immunol..

[112] Glienke W., Esser R., Priesner C., Suerth J.D., Schambach A., Wels W.S., Grez M., Kloess S., Arseniev L., Koehl U. (2015). Advantages and applications of CAR-expressing natural killer cells. Front. Pharmacol..

[113] Roth T.L., Puig-Saus C., Yu R., Shifrut E., Carnevale J., Li P.J., Hiatt J., Saco J., Krystofinski P., Li H. (2018). Reprogramming human T cell function and specificity with non-viral genome targeting. Nature.

[114] Chen Z.H., Yu Y.P., Zuo Z.H., Nelson J.B., Michalopoulos G.K., Monga S., Liu S., Tseng G., Luo J.H. (2017). Targeting genomic rearrangements in tumor cells through Cas9-mediated insertion of a suicide gene. Nat. Biotechnol..

